# Implementation of a monitoring and contact tracing program to manage COVID-19 by a multinational chemical company

**DOI:** 10.47626/1679-4435-2023-1109

**Published:** 2023-11-24

**Authors:** Daniel Astun Cirino, Henrique Ceretta Oliveira, Ariane Polidoro Dini, Marcia Bandini

**Affiliations:** 1 Departamento de Saúde Coletiva, Faculdade de Ciências Médicas, Universidade Estadual de Campinas (Unicamp), Campinas, SP, Brazil; 2 Faculdade de Enfermagem, Unicamp, Campinas, SP, Brazil

**Keywords:** COVID-19, pandemics, epidemiology, contact tracing, occupational health, COVID-19, pandemias, epidemiologia, busca de comunicante, saúde do trabalhador

## Abstract

**Introduction:**

During the SARS-CoV-2 pandemic, uninterrupted manufacture of products for domestic or health care purposes presupposed initiatives to control transmission in the work environment.

**Objectives:**

This study analyzed data collected in a multinational chemical company between 2020 and 2022 through its COVID-19 contact tracing system, determining the association between work variables, personal protective equipment use, emotional distress symptoms, and diagnostic confirmation of COVID-19.

**Methods:**

This correlational, cross-sectional study analyzed a company database of reports of suspected cases between June 2020 and January 2022. Descriptive analysis was performed, and the chi-square test was used to study the associations between the variables, with a significance level of 5%.

**Results:**

Of the 4206 total reports, 1190 diagnoses of COVID-19 were confirmed. The following variables were associated with infection: age over 40 years, being symptomatic, being a remote worker, and reporting difficulties with emotional control.

**Conclusions:**

The results identified the potential of on-the-job education activities, as well as that control and prevention measures protected worker health, and that worker mental health should be monitored.

## Abstract (pt)

**Introdução:**

O trabalho ininterrupto de profissionais que atuaram na produção de insumos para uso doméstico ou para a assistência à saúde pressupôs ações para o controle da transmissão do SARS-CoV-2 no ambiente de trabalho e, assim, prevenir o adoecimento dos trabalhadores.

**Objetivos:**

Descrever os dados resultantes do rastreamento de contatos para covid-19; identificar condições de sofrimento emocional no período de 2020-2022; e verificar a associação entre variáveis laborais, uso de equipamento de proteção individual, sintomas de sofrimento emocional e a confirmação diagnóstica de covid-19 em trabalhadores de uma indústria química multinacional.

**Métodos:**

Estudo correlacional, de corte transversal. Foi analisado um banco de dados composto pelos registros de casos suspeitos entre junho de 2020 e janeiro de 2022 de uma empresa multinacional. Foi realizada análise descritiva e, para estudar as associações entre as variáveis, foram aplicados os testes qui-quadrado, sendo considerado um nível de significância igual a 5%.

**Resultados:**

Dos 4.206 registros realizados, 1.190 respostas apresentaram resultado positivo de covid-19. Profissionais maiores de 40 anos, sintomáticos, em atividade remota e que declaram dificuldades para controlar sentimentos apresentaram maior positividade para covid-19.

**Conclusões:**

Identificou-se a potencialidade das atividades de educação em serviço presencial e que a fiscalização de medidas de controle e prevenção protegeu a saúde do trabalhador. Em relação à saúde mental, os resultados indicaram a necessidade de acompanhamento dos trabalhadores para identificar potenciais agravos e sofrimentos mentais decorrentes da pandemia.

## INTRODUCTION

The COVID-19 pandemic greatly impacted the world of work through measures to control transmission, provide safety, and protect worker health. Factories adapted their production spaces and common areas, implementing new rules, procedures, control measures, and equipment usage.^1-3^

Being an infectious disease transmitted primarily through respiratory droplets during close in-person contact, preventing the transmission of COVID-19 in the workplace is largely based on the following strategies: physical distancing, constant mask usage, greater ventilation, hygienization of hands and workplaces, self-observation for signs and symptoms, identification of suspected cases for isolation and monitoring, and testing for diagnostic confirmation.^4,5^

Contact tracing/mapping was an important procedure for controlling the spread of COVID-19 since it proactively identified potentially infected individuals, whether symptomatic or asymptomatic, allowing company occupational health services to refer them for isolation or quarantine, thus combating transmission.^6,7^

The emergence of new variants of the SARS-CoV-2 virus, the occurrence of sequential epidemic outbreaks (popularly known as “new waves”) and the unequal distribution of COVID-19 vaccines, as well as raw material restrictions that resulted in production shortfalls, created a complex and challenging global scenario for satisfactory pandemic control.^1-3,5,7^

The economic and social crises surrounding the pandemic have made it difficult for companies to maintain operations, providing employment and income to workers in a context of greater volatility, uncertainty, complexity, and ambiguity (VUCA) and in the global market.^6,8,9^ The term “VUCA” was coined by the U.S. Army War College in the late 1980s in reference to post-Cold War geopolitics, and its use in the corporate environment has been renewed during extraordinary situations, such as the recent pandemic.^8,9^

The production of personal protective masks, respirators, health care materials, and household products, which are critical even for those able to remain isolated during the COVID-19 pandemic, has continued uninterrupted. However, controlling the transmission of SARS-CoV-2 in the workplace is a collective health initiative that presupposes managerial initiatives to promote health and prevent worker illness.^1,5^

This study investigated workplace epidemiological surveillance and strategies to control the spread of COVID-19, analyzing a multinational chemical industry’s contact tracing data for COVID-19 between 2020 and 2022, verifying the association between work variables, personal protective equipment use, emotional distress symptoms, and diagnostic confirmation of COVID-19 in workers.

## METHODS

This quantitative, correlational, cross-sectional study followed the Strengthening the Reporting of Observational Studies in Epidemiology (STROBE) guidelines for observational studies. It was conducted in a multinational corporation that develops essential products for society and, within the scope of legislation regarding the COVID-19 pandemic, production was maintained to avoid shortages. The company’s diverse product range encompasses health care, personal protective equipment, consumer goods, food safety, and the transportation, electronics, automotive, and aerospace industries. During the pandemic, it manufactured high-performance masks for respiratory protection, known as filtering facepiece respirators. In Brazil, where the study was conducted, the company has 3,300 permanent employees and approximately 2,000 subcontracted workers.

Data was collected from a corporate occupational medicine database developed prior to the study to monitor suspected COVID-19 cases among workers. The company’s legal department authorized our use of the database for research purposes. This research project was approved the institutional research ethics committee (5,198,871). After ethical approval, the database was analyzed, excluding data that could identify individual workers. This study complied with Brazilian Resolution 466/12, which concerns research involving human beings.

The database consisted of reports of suspected cases occurring between June 2020 and January 2022, including the following variables: age, sex, symptoms, work characteristics, respirator use, potential exposure to SARS-CoV-2 in the workplace (by describing close contact with suspected cases), work model (remote, hybrid, or in-person), work shift, and confirmed COVID-19 diagnosis.

The data were exported to Microsoft Excel spreadsheets. Pearson’s Chi-square test was used to determine the associations between qualitative variables and confirmed COVID-19 diagnosis. A significance level of 5% was used for all analyses.^10^

## RESULTS

The database included reports of 4206 suspected cases between June 2020 and January 2022. It should be pointed out that the database consists of responses rather than respondents, ie, one worker could have contributed multiple reports.

The most common worker age range was 30-39 years (39.9%), followed by 20-29 years (25.2%), 40-49 years (23.9%), 50-59 years (7.5%), <20 years (2.2%), and >60 years (0.2%). Regarding plant location, 92.9% of the reports were from workers in the state of São Paulo (Plants 1, 2, 3, 5 and 6) and 7.1% from the Manaus plant (Plant 4). Among the 4206 workers, the most prevalent shifts, in descending order, were: morning (31.9%), administrative (28.6%), afternoon (25.6%), night (12.2%), and 12-for-36 (ie, 12-hour workdays on alternate days) (1.7%).

The work models during the pandemic, in descending order of prevalence, were in-person (80.9%), hybrid (12.1%) and remote (7%). There were 3 employment types: hourly (61%), ie, those directly involved in industrial production; monthly (16.6%), ie, those with predominantly administrative activities; and outsourced (22.5%), ie, service providers and suppliers. There was a higher prevalence of hourly and in-person workers (Table 1).

**Table 1:** Distribution of sociodemographic and work-related characteristics, Brazil, 2020-2022

Variable	Answers	
	n	%
Age (years)		
≤ 19	91	2.2
20-29	1,062	25.2
30-39	1,680	39.9
40-49	1,004	23.9
50-59	317	7.5
≥ 60	8	0.2
No data	44	1.1
Company branches in Brazil		
Plant 1	1,798	42.8
Plant 2	1,315	31.3
Plant 3	559	13.3
Plant 4	299	7.1
Plant 5	212	5.0
Plant 6	23	0.5
Work shift		
Morning	1,341	31.9
Evening	1,075	25.6
Night	512	12.2
Administrative	1,205	28.6
12 x 36	73	1.7
Work format during the pandemic		
In person	3,405	81.0
Remote	508	12.0
Hybrid	293	7.0
Type of employment contract		
Hourly	2,562	60.9
Monthly	700	16.6
Outsourced	944	22.5

Regarding the tracing and monitoring of COVID-19 among workers, adherence to preventive protocols was quite high (98.4%), and there was a low frequency of risky situations, such as close contact in the work environment (2.9 %) (Table 2). Among all 1190 responses during the study period, 28.3% corresponded to workers who tested positive for COVID-19.

**Table 2:** COVID-19 tracing and monitoring characteristics, Brazil, 2020-2022

Variable	Answers	
	n	%
N-compliance with preventive protocols		
No	4,139	98.4
Yes	67	1.6
COVID-19 diagnosis		
Negative	3,016	71.7
Positive	1,190	28.3
Close contact in the workplace		
No	3,766	89.5
Yes	123	2.9
No data	317	7.6

**Figure 1:**
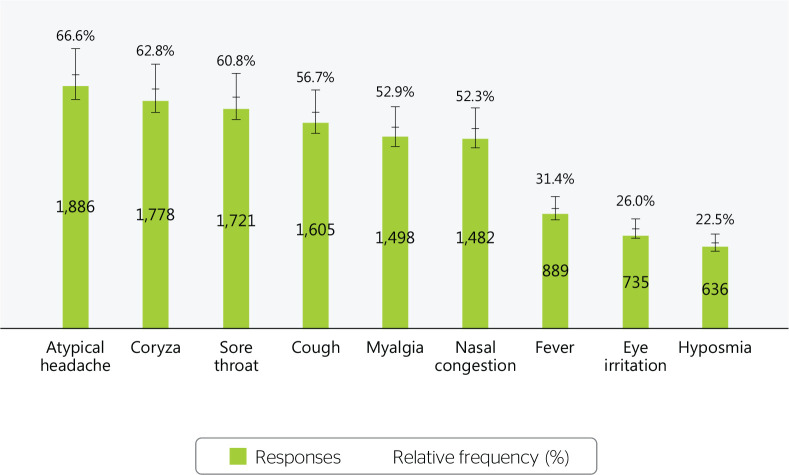
Main symptoms reported in contact tracing, Brazil, 2020-2022.

The main symptoms mentioned in the contact tracing interview were headache (66.6%), runny nose (62.8%), sore throat (60.8%), cough (56.7%), body pain (52.9%), nasal congestion (52.3%), fever (31.4%), eye irritation (26%), and reduced sense of smell or taste (22.5%) (Figure 1).

**Figure 2:**
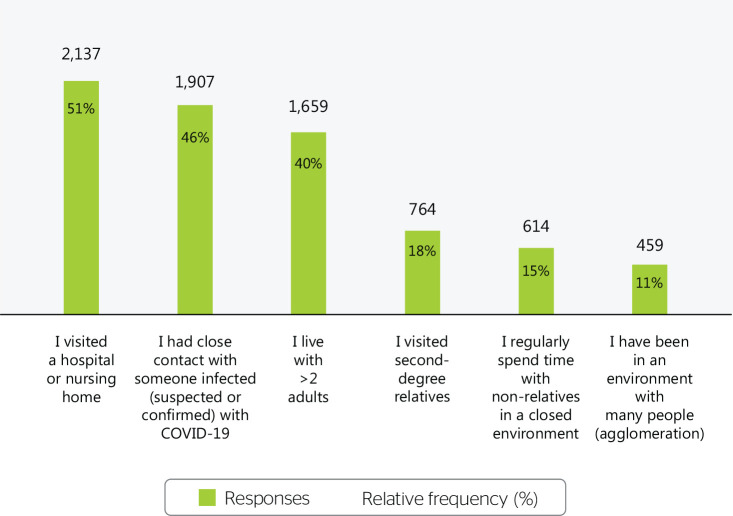
COVID-19 exposure risk outside the work environment, Brazil, 2020-2022.

The main community risk situations for COVID-19 infection were: visiting a hospital or nursing home (51.6%), close contact with a sick person (46%), living with >2 adults (40%), visiting second-degree relatives (18.4%), regularly spending time with non-relatives in a closed environment (14.8%), and being in a crowd (11.1%) (Figure 2).

**Figure 3:**
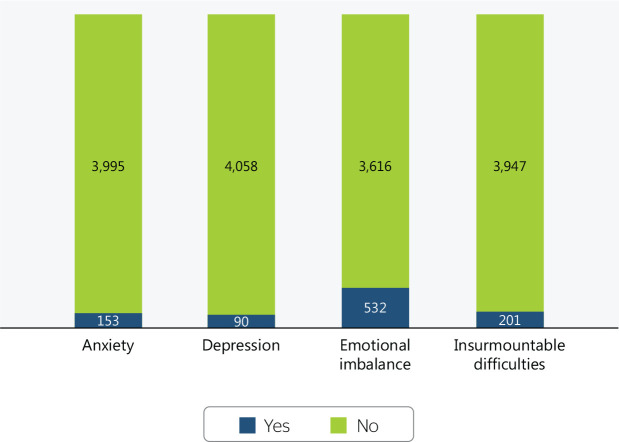
Worker self-perceived emotional state, Brazil, 2020-2022.

Regarding self-perceived emotional state, 12.8% (n = 532) reported some difficulty controlling their feelings due to unforeseen events in everyday life and 4.8% (n = 201) reported feeling that their difficulties were becoming insurmountable (Figure 3).

Workers aged < 30 years were the least infected group, while those aged > 40 years had the highest proportion of diagnosed COVID-19 cases. There was a higher proportion of COVID-19 infection among remote workers than in-person or hybrid workers. Use of a filtering facepiece respirator while working had a protective effect, ie, those who used one had a lower chance of COVID-19 infection. Symptomatic workers who had contact with someone with a diagnosed COVID-19 infection had a higher infection rate than symptomatic workers who did not and a higher infection rate than asymptomatic workers. Workers who reported difficulties controlling their feelings and reactions to unforeseen events had a higher proportion of COVID-19 infection than those who did not report such difficulties (Table 3).

**Table 3:** Association between variables and COVID-19 diagnosis, Brazil, 2020-2022

Variable	COVID-19 diagnosis				p-value^*^
	Negative		Positive		
	n	%	n	%	
Age (years)					<0.0001
≤29	887	76.93	266	23.07	
30-39	1,202	71.55	478	28.45	
40-49	672	66.93	332	33.07	
≥50	228	69.94	98	30.06	
Work model					<0.0001
Remote	276	54.33	232	45.67	
In person/hybrid	2,740	74.09	958	25.91	
Type of employment contract					<0.0001
Hourly	1,862	72.68	700	27.32	
Monthly	428	61.14	272	38.86	
Outsourced	726	76.91	218	23.09	
N95 respirators used in work activity?					<0.0001
No	647	64.00	364	36.00	
Yes	2,312	74.51	791	25.49	
Close contact with a positive COVID-19 case in the last 2 weeks?					<0.0001
No	2,400	73.42	869	26.58	
Yes	610	65.95	315	34.05	
Have you had any COVID-19-related symptoms in the last 15 days?					<0.0001
No	1,049	77.47	305	22.53	
Yes	1,965	69.00	883	31.00	
Difficulty controlling feelings and reactions to unforeseen events?					
Never/rarely	2,875	72.18	1,108	27.82	0.0105
Frequently/always	104	63.03	61	36.97	

* Chi-square test.

## DISCUSSION

Workers who participated in the contact tracing process were predominantly between 30 and 39 years of age (40% of the overall reports; 28% of the confirmed COVID-19 cases), although there was a higher proportion of cases among workers aged 40-49 years (24% of the overall reports; 33% of the confirmed COVID-19 cases).

According to chronic non-communicable disease prevalence data, systemic arterial hypertension and diabetes mellitus occur 18% and 4% of Brazilians aged 35 to 44 years, respectively, and in 34% and 8% of those aged 45 to 54 years, respectively.^11^ Considering that COVID-19 can increase the risk of worsening comorbidities,^11,12^ in addition to the cardiovascular clinical repercussions of long COVID-19, suspected case monitoring and contact tracing programs must pay special attention to workers aged 40 to 49 years.

Regarding work characteristics, 88% of the reports were from in-person or hybrid model workers (≥2 days of in-person work per week) workers, ie, the bulk of those who used company facilities and were subject to monitoring, given that the plants were considered high-risk locations for SARS-CoV-2 outbreaks and subsequent community transmission.^13^ Another important fact is that 22% of the population was outsourced workers (service providers), which indicates that all workers who operated in the same work environment were included without distinction according to employment relationship, revealing the thorough level of support and epidemiological coverage.^13^

Regarding adherence to preventive protocols, 123 potential exposure events were reported in the work environment, as opposed to 1907 such events outside the work environment, the majority of which involved health care-related exposure (51% in visits to hospitals or nursing homes), housing (living with >2 adults in the same house), and family visits.

The interesting results for self-perceived emotional state (>95% reported no emotional distress; see Figure 3) may have been due to the following factors, which could positively affect mental health: the availability of personal protective equipment, training, a positive work environment (trust), and emotional support.^14,15^

There was a positive association between lower relative frequency of COVID-19 diagnosis (25%) and mandatory use of filtering facepiece respirators as personal protective equipment compared to work activities for which their use was merely recommended (36%). This result corroborates the efficiency of high-performance respirators for controlling transmission, as previously demonstrated by the U.S. Centers for Disease Control and Prevention.^16^

The results suggest that in-person work is safer than remote work (26% vs 46% of the infections), which indicates how the work environment can positively affect adherence to preventive protocols through collective control and prevention measures (such as ventilated spaces and physical distancing). The results also demonstrate the need for greater support for remote workers, as well as the effects of pandemic fatigue, which contributes to flexible behavior regarding infection risk.^17^

The contact tracing program involved identifying, minimizing, and monitoring contact with suspected and/or confirmed cases of COVID-19.^5,18^ Active searching was selected to facilitate adherence to this process, thus epidemiological surveillance was an important instrument for increasing communication and promoting initiatives to preserve and improve organizational performance.

In the context of worker health management, considering that quarantining millions of people simultaneously with no foreseeable end in sight had never been done before, the surveillance process included questions on emotional well-being to monitor negative aspects of mental health.^19^ Although the reported symptoms of mental suffering were not overly worrying, it is important to point out that data collection was not anonymous for these questions and, thus, social desirability bias must be considered, ie, respondents may have downplayed mental health symptoms.^20^ Therefore, ways to increase our understanding of the true psychological impact of the pandemic warrant further consideration.^19^

Study limitations included the cross-sectional design, which cannot identify cause and effect, as well as the fact that the first author is a medical manager at the data collection site. Regarding the potential for future research, analyzing the work environment as a protective factor for worker health is clearly relevant.

## CONCLUSIONS

A data collection process was implemented for contact tracing that transcended the context of the pandemic, covering topics such as mental health and the safety of the work environment. This data provided a basis for initiatives to promote health in the workplace. According to the results, there was a lower percentage of confirmed COVID-19 infection among in-person workers than remote workers. This indicates the potential of educational activities for in-person work, as well as that of safety measures, such as high-performance respiratory protective equipment, given that modifying the work environment through control and prevention measures protected worker health. However, the results also indicated opportunities for training among remote workers.

The mental health results were inconclusive and indicate the need for professional monitoring to identify potential mental health problems and suffering due to the pandemic.

## References

[1] Koh D, Goh HP. (2020). Occupational health responses to COVID-19: what lessons can we
learn from SARS?. J Occup Health.

[2] Cucinotta D, Vanelli M. (2020). WHO declares COVID-19 a pandemic. Acta Biomed.

[3] Zhu N, Zhang D, Wang W, Li X, Yang B, Song J (2020). A novel coronavirus from patients with pneumonia in China,
2019. N Engl J Med.

[4] Aquino EML, Silveira IH, Pescarini JM, Aquino R, Souza-Filho JA, Rocha AS (2020). Social distancing measures to control the COVID-19 pandemic:
potential impacts and challenges in Brazil. Cien Saude Colet.

[5] Kniffin KM, Narayanan J, Anseel F, Antonakis J, Ashford SP, Bakker AB (2021). COVID-19 and the workplace: implications, issues, and insights
for future research and action. Am Psychol.

[6] Calvo RA, Deterding S, Ryan RM. (2020). Health surveillance during covid-19 pandemic. BMJ.

[7] Dehghani F, Omidi F, Yousefinejad S, Taheri E. (2020). The hierarchy of preventive measures to protect workers against
the COVID-19 pandemic: a review. Work.

[8] Bennett N, Lemoine GJ. (2014). What a difference a word makes: understanding threats to
performance in a VUCA world. Bus Horiz.

[9] Whiteman WE. (1998). Training and educating army officers for the 21st century: implications
for the United States Military Academy.

[10] Pagano M, Gauvreau K. (2004). Princípios de bioestatística.

[11] Brasil, Ministério da Saúde, Secretaria de Vigilância em Saúde, Departamento de Análise em Saúde e Vigilância
de Doenças No Transmissíveis (2022). Vigitel Brasil 2006-2020: morbidade referida e
autoavaliação de saúde. Vigilância de fatores de
risco e proteção para doenças crônicas por
inquérito telefônico: estimativas sobre frequência e
distribuição sociodemográfica de morbidade referida e
autoavaliação de saúde nas capitais dos 26 estados
brasileiros e no Distrito Federal entre 2006 e 2020.

[12] Ejaz H, Alsrhani A, Zafar A, Javed H, Junaid K, Abdalla AE (2020). COVID-19 and comorbidities: deleterious impact on infected
patients. J Infect Public Health.

[13] Ingram C, Downey V, Roe M, Chen Y, Archibald M, Kallas KA (2021). COVID-19 prevention and control measures in workplace settings: a
rapid review and meta-analysis. Int J Environ Res Public Health.

[14] Khajuria A, Tomaszewski W, Liu Z, Chen JH, Mehdian R, Fleming S (2021). Workplace factors associated with mental health of healthcare
workers during the COVID-19 pandemic: an international cross-sectional
study. BMC Health Serv Res.

[15] Bulińska-Stangrecka H, Bagieńska A. (2021). The role of employee relations in shaping job satisfaction as an
element promoting positive mental health at work in the era of
COVID-19. Int J Environ Res Public Health.

[16] Andrejko KL, Pry JM, Myers JF, Fukui N, DeGuzman JL, Openshaw J (2022). Effectiveness of face mask or respirator use in indoor public
settings for prevention of SARS-CoV-2 Infection - California,
February-December 2021. MMWR Morb Mortal Wkly Rep.

[17] Petherick A, Goldszmidt R, Andrade EB, Furst R, Hale T, Pott A (2021). A worldwide assessment of changes in adherence to COVID-19
protective behaviours and hypothesized pandemic fatigue. Nat Hum Behav.

[18] Breeher L, Boon A, Hainy C, Murad MH, Wittich C, Swift M. (2020). A framework for sustainable contact tracing and exposure
investigation for large health systems. Mayo Clin Proc.

[19] Afonso P. (2020). The Impact of the COVID-19 pandemic on mental
health. Acta Med Port.

[20] Bergen N, Labonté R. (2020). “Everything is perfect, and we have no problems”: detecting and
limiting social desirability bias in qualitative research. Qual Health Res.

